# Exploring the Connection between *Porphyromonas gingivalis* and Neurodegenerative Diseases: A Pilot Quantitative Study on the Bacterium Abundance in Oral Cavity and the Amount of Antibodies in Serum

**DOI:** 10.3390/biom11060845

**Published:** 2021-06-06

**Authors:** Raffaella Franciotti, Pamela Pignatelli, Claudia Carrarini, Federica Maria Romei, Martina Mastrippolito, Antonella Gentile, Rosa Mancinelli, Stefania Fulle, Adriano Piattelli, Marco Onofrj, Maria Cristina Curia

**Affiliations:** 1 Department of Neuroscience, Imaging and Clinical Sciences, “G. d’Annunzio” University of Chieti-Pescara, 66013 Chieti, Italy; raffaella.franciotti@unich.it (R.F.); claudia.carrarini@live.it (C.C.); r.mancinelli@unich.it (R.M.); stefania.fulle@unich.it (S.F.); onofrj@unich.it (M.O.); 2 Department of Medical, Oral and Biotechnological Sciences, “G. d’Annunzio” University of Chieti-Pescara, 66013 Chieti, Italy; pamelapignatelli89p@gmail.com (P.P.); federicamaria.romei@gmail.com (F.M.R.); martina.mastrippolito@studenti.unich.it (M.M.); antogent@virgilio.it (A.G.); apiattelli@unich.it (A.P.); 3 Fondazione Villa Serena per la Ricerca, Città S. Angelo, 65013 Pescara, Italy; 4 Casa di Cura “Villa Serena” del Dott. L. Petruzzi, Città S. Angelo, 65013 Pescara, Italy

**Keywords:** oral bacteria, periodontitis, *Porphyromonas gingivalis*, neurological disease, neurodegenerative disease, genomic DNA, antibody

## Abstract

Recent studies support the hypothesis that microbes can seed some Alzheimer’s disease (AD) cases, leading to inflammation and overproduction of amyloid peptides. *Porphyromonas gingivalis* (Pg) is a keystone pathogen of chronic periodontitis and has been identified as risk factor for the development and progression of AD. The present preliminary study aimed to quantify Pg abundance in neurodegenerative disease (ND) patients compared with neurologic patients without neurodegenerative disorders (no-ND) and healthy controls (HC) to determine possible association between Pg abundance and neurodegenerative process. Pg was quantified on DNA extracted from the oral samples of 49 patients and 29 HC by quantitative polymerase chain reaction (qPCR). Anti-Pg antibodies were also detected on patient serum samples by enzyme-linked immunosorbent assays (ELISA). The Pg abundance in the oral cavity was significantly different among groups (*p* = 0.004). It was higher in ND than no-ND (*p* = 0.010) and HC (*p* = 0.008). The Pg abundance was correlated with the antibodies (*p* = 0.001) with different slopes between ND and no-ND (*p* = 0.037). Pg abundance was not correlated with oral indices and comorbidities. These results extend our understanding of the association between oral pathogens and AD to other neurodegenerative processes, confirming the hypothesis that oral pathogens can induce an antibody systemic response, influencing the progression of the disease.

## 1. Introduction

The theory that an infection can contribute to the onset of Alzheimer’s Disease (AD), a progressive neurodegenerative disorder has been held for many years among researchers. This theory is becoming increasingly recognized [1,2,3], suggesting that microbes can lead to the overproduction of sticky and soluble proteins in the brain, amyloid-β peptides [4], which clump into plaques and may cause neuroinflammation [5]. Altered gene expression induces the release of pro-inflammatory cytokines that stimulate the production of highly reactive oxygen and nitrogen species (ROS and RNS, respectively), resulting in an impairment in the surrounding tissues and activating surrounding glial cells [6]. Brain cell atrophy, immunological aberrations, amyloidogenesis, and cognitive deficits are also consequences of microbial infection [7,8,9,10]. Until recently, it was mostly thought that resident oral bacteria were only capable of generating disease confined within the oral cavity. However, recent studies have demonstrated that the oral microbiome, in addition to the gut microbiome, plays a role in the onset of neuroinflammation in neurodegenerative diseases. Bacterial lipopolysaccharides (LPS) activate Toll-like receptors (TLRs) expressed in glial cells, inducing an inflammatory response due to the overexpression of pro-inflammatory cytokines such as IL-6, IL-1,TNF-α, and IFN-γ [11]. Both intestinal and oral microbes can pass into the blood, and it was hypothesized that some pathogens can directly cross a weakened blood–brain barrier, reaching the central nervous system and causing neurological damage. The microorganisms that enter the blood and circulate throughout the body are usually promptly eliminated by the reticuloendothelial system (transient bacteremia). However, if they find favorable conditions and, subsequently, increased permeability of the barriers, they may settle in a given site and produce infection-induced inflammation.

The microbiota can indirectly affect brain function by modulating the kynurenine pathway (KP), influencing the production of tryptophan and the degradation of neuroactive compounds, and finally the production of kynurenines bioactive metabolites with neurotoxic and immunologic activities [6,12].

Periodontitis is a multifactorial, inflammatory disease promoted by the dysbiosis of the oral biofilm that can lead to tooth loss in the later stages of the disease [13]. It was recently found that in individuals with cognitive impairment or AD, the subgingival microbiota shows changes typical of periodontal disease [14], and tooth loss was associated with cognitive and physical deterioration [15].

Periodontitis is accompanied by a systemic antibody response against periodontal pathogens, which can be conveniently measured with an immunoassay. Like other common infectious diseases, serum levels of antibodies against periodontal pathogens can therefore be useful markers for the detection of systemic diseases. The presence of serum antibodies to major periodontal pathogens has been already associated with heart disease and stroke [16,17,18]. Then, an antibody test against periodontal bacteria may be effective in assessing the effect of periodontal infection on systemic disease.

The keystone pathogen responsible for the onset of chronic periodontal disease is *Porphyromonas gingivalis* (Pg) [19]. Numerous studies have suggested that Pg enhances the pathogenesis of adverse pregnancy outcomes [20], rheumatoid arthritis [21], and atherosclerotic cardiovascular disease [22]. Pg subverts the host immune system response, invades human epithelial and endothelial cells, stimulates cell proliferation and promotes carcinoma cell migration by inhibiting the p53 tumor suppressor, and alters the homeostasis of the entire oral biofilm, enhancing the pathogenicity of a polymicrobial community [15]. Local inflammation is triggered by the interaction between the host immune response and bacterial biofilm load [23], which may lead directly or indirectly to a state of chronic low-grade systemic inflammation.

Pg was also associated with impaired spatial/episodic memory in AD [24] and it is a candidate pathogen as co-factor for the development of neurological diseases through circulatory or neural access to the brain due to transient bacteremia and inflammatory mediators [25,26].

Both Pg and its virulence products, such as fimbrins, gingipain, and LPS of the outer membranes, can enter the bloodstream, promoting the expression of cytokines, prostaglandins, and growth factors [27]. The spread of Pg from the oral cavity to other sites is probably due to the formation of circulating outer membrane vesicles (OMVs), leading to secondary non-oral diseases [11]. Pg has been observed away from the oral cavity in atherosclerotic carotid plaques [28], in placenta and fetal tissues of rats [29], and in post-mortem cerebral tissue samples taken from AD patients [3].

At present, the abundance of Pg in the oral cavity and the anti-Pg antibodies in the serum have been little studied in patients with neurodegenerative disease.

Serum IgG antibodies against periodontal pathogens have been especially associated with AD [30,31]. In particular, elevated levels of immunoglobulin G (IgG) against Pg were detected in subjects prior to cognitive impairment, indicating an involvement of this bacterium in cognitive decline [32,33].

The aim of the study was to investigate if the abundance of Pg in the oral cavity is associated with neurodegenerative diseases and with the presence of anti-Pg antibodies in the serum. We also analyzed the possible relationships between Pg quantity, serum antibodies, and clinical characteristics (inflammatory and metabolic markers) of neurological patients suffering from neurodegenerative and non-neurodegenerative diseases.

## 2. Materials and Methods

### 2.1. Study Cohort

Neurological patients, referred to the Neurology Clinic of “SS Annunziata” Hospital of Chieti, were enrolled, once a week, from May 2020 to March 2021. Written informed consent was obtained from 55 patients, who were enrolled in the study.

As control group, we recruited 30 healthy controls (HC) free from neurological diseases from a list of healthy volunteers.

From the enrolled participants, individuals under antibiotic therapy or using daily chlorhexidine mouthwash within the last 3 months or with a diagnosis were excluded. The final cohort included 21 patients suffering from neurodegenerative disease (ND), 28 patients who received different diagnoses classified as non-neurodegenerative diseases (no-ND), and 29 HC.

In the ND group, 8 (38.1%) patients had AD [34]; 3 (14.3%) belonged to a frontotemporal dementia (FTD) spectrum [35,36,37,38]; 7 (33.3%) had a diagnosis of parkinsonism, which included 1 Parkinson’s disease (PD) [39], 3 PD with dementia (PDD) [40], and 3 dementia with Lewy bodies (DLB) [41]; 2 (9.5%) had multiple sclerosis (MS) [42]; and 1 (4.8%) patient suffered from Huntington disease (HD) [43]. In addition, for all AD and FTD patients, brain magnetic resonance imaging (MRI), neuropsychological assessment, and lumbar puncture procedure (or eventually a brain positron emission tomography scan) were performed. Two out of three FTD patients also underwent an electromyography exam. PD, PDD, and DLB patients were diagnosed by a clinical and neuropsychological evaluation according to international diagnostic criteria. All MS patients underwent both brain MRI and a lumbar puncture procedure. The diagnosis of HD was confirmed by a genetic test after a clinical and familial evaluation.

The group of no-ND included: 10 (35.7%) patients with cerebrovascular disease (3 individuals with acute stroke, 2 with intracranial hemorrhage, and 5 with vascular dementia), 4 (14.3%) with encephalitis, 3 (10.7%) with epilepsy, 2 (7.1%) with persistent headache, 2 (7.1%) with myelopathy, and 7 (25%) with a peripheral nervous system (PNS) disorder (4 subjects with myasthenia gravis and 3 with a polyneuropathy).

### 2.2. Oral Examination

All participants underwent a complete assessment of oral health status. Oral examinations were performed by a trained dentist (P.P) using mirrors (MIR3HD, Hu-Friedy, Chicago, IL, USA), a dental probe (PCP-UNC 15, Hu-Friedy, Chicago, IL, USA), and an intra-oral light.

During the oral examination, teeth number, plaque index (PI), and gingival index (GI) were recorded using the standardized Oral Health Questionnaire. Briefly, the PI and GI were measured at six surfaces (buccal-mesial, mid-buccal, buccal-distal, lingual-mesial, mid-lingual, and lingual-distal) on the Ramfjord teeth (the maxillary right first molar, maxillary left central incisor, maxillary left first premolar, mandibular left first molar, mandibular right central incisor, and mandibular right first premolar) with a manual probe [44]. The presence of gingivitis and oral infection was tabulated with a value of 1 if gingival bleeding, abscesses or residual roots were present; and with a value of 0 if absent. The presence of a double, only upper/lower, or absence of mobile prosthesis was recorded with an index from 2 to 0, respectively. Similar to the preceding index, the occurrence of multiple, single, or absence of oral implant/crown was scored with an index from 2 to 0, respectively.

The Oral Health Index was used to indicate good (scored as 2), sufficient (scored as 1), and insufficient (scored as 0) hygiene, evaluating the amount of dental plaque, supragingival calculus, and oral food debris. The hygiene status of the tongue was assessed through the Lingual Patina Index as: 0, no visible patina on the dorsum of the tongue; 1, presence of patina on the posterior third of the back of the tongue; 2, presence of patina all over the back of the tongue, which does not cover the mucosal color; and 3, presence of patina all over the back of the tongue, thick masking the mucosa of the tongue. 

### 2.3. Collection of the Samples

Tongue biofilm was taken from each patient and each control subject by the same dentist (P.P.) under the same conditions, 8 h after the last brushing of teeth. The swab was collected by brushing 5 times from the middle third of the tongue dorsum. After shaking it vigorously for 30s, the swab was immediately transferred into 5.0 mL of phosphate-buffered saline (PBS) and then kept at 4 °C until nucleic acid extraction. A blood sample was also taken from 37 patients (15 from ND patients and 22 from no-ND patients) on the same day of the brushing and stored at −80 °C.

### 2.4. Evaluation of Comorbidities in Patients

Routine biochemistry tests were collected from patients’ records. Specifically, the inflammatory markers (i.e., C-reactive protein (CRP), white blood cell count (WBC), neutrophil count (NC), lymphocytes count (LC)), metabolic markers (i.e., total cholesterol (TC), high-density lipoprotein (HDL), triglycerides (TGs)), hypertension, and diabetes) were gathered. Then, systemic inflammation was considered present if CRP > 5.00 mg/L, WBC > 10.00 × 10^3^/µL, NC > 7.10 × 10^3^/µL, and/or LC > 3.00 × 10^3^/µL). For the cholesterol marker, the ratio TC/HDL was considered. Abnormal cholesterol and high triglycerides values were associated with TC/HDL > 4.5 and TGs > 150 mg/dL, respectively. Hypertension and diabetes were considered present when blood pressure was greater than 140/90 mmHg and glycemia was >126 mg/dL. For ND and no-ND groups, the percentage of patients showing each comorbidity was calculated and then compared statistically.

### 2.5. DNA Isolation and Bacterial DNA Quantification on Brushing

Pg ATCC 33277 (LGC Standards S.r.l., Sesto San Giovanni, Milano, Italy) was the bacterial strain used in this study. The bacterial strain growth conditions were previously reported [45]. Molecular analyses were performed to quantify the Pg bacterial strain in all participants to assess the presence of any imbalances in the oral microbial flora. Total genomic DNA was isolated from samples and from bacterial strain using aQuick DNA miniPrep Plus KIT (Zymo Research, Cambridge Bioscience, UK). qPCR analysis to quantify Pg abundance in our samples was performed using StepOne™ 2.0 (Applied Biosystems, Thermo Fisher Scientific, Waltham, MA, USA). A TaqMan-based assay that recognizes Pg 16S rRNA, the gene encoding the small subunit of 16S ribosomal RNA, was used as previously reported [45]. A standard curve passing through 5 points was constructed indicating the cycle threshold values versus the Pg 16S rRNA gene. Based on this method, it was possible to estimate the bacterial quantity related to the amount of total DNA isolated from the oral samples. Pg DNA concentrations used as standard were previously published [45].

### 2.6. Antibody Assay of Serum

Serum IgG antibody responses to bacterial pathogens from Pg were assayed by ELISA using the ChonBlock^TM^ buffer system (Human Anti-Bacteria & Toxins Antibody ELISA Kits, #6119, an ELISA protocol to improve the accuracy and reliability of serological antibody assays, Chondrex, Woodinville, WA, USA) as described in detail previously [46]. The assay was performed at room temperature using specific 96-well plates. For dilution, samples less than 1:1000 were run on both antigen-coated and uncoated plates; for dilution samples more than 1:1000 were run only on antigen-coated plates. Briefly, sera from participants collected and stored at -80 °C were thawed and centrifuged at 10,000 rpm for 5 min. The supernatants were collected and appropriately diluted with ChonBlock^TM^ standard/sample dilution buffer. Briefly, blocking buffer was added to each well and incubated for 1 h; the plate was washed using wash buffer; standards and samples were added and incubated for 2 h; the plate was washed using wash buffer; secondary antibody solution (ChonBlock^TM^ ELISA Kit, Chondrex, Woodinville, USA) was added and incubated for 1 h; the plate was washed using wash buffer; TMB solution was added and incubated for 25 min; stop solution was added; and the OD values at 450 nm were recorded within 5 min. Antibody levels were determined by comparing them to standard levels and analyzed using regression analysis. The antibody levels are expressed as EU (10^3^ units/mL).

### 2.7. Statistical Analyses

Data are reported as mean ± standard deviation (SD) or percentage for continuous and dichotomous variables, respectively. Continuous data were compared between groups using analysis of variance (ANOVA). The Bonferroni test was applied for the post hoc comparisons. Nonparametric statistics were applied for the comparison of dychotomous variables. The Kruskal–Wallis test was applied for the post hoc analyses. Spearman’s correlation test was performed to evaluate the association between the variables. The level of significance was set to 0.05.

## 3. Results

We evaluated the abundance of Pg in the oral cavity of 49 patients suffering from neurological disease, 21 affected by ND and 28 affected by no-ND, and in 29 HC by qPCR. We also assessed the presence of anti-Pg antibodies in the patients’ sera.

### 3.1. Clinical and Oral Outcomes

Demographic characteristics and oral indices of the groups are shown in Table 1.

Demographic characteristics (i.e., age and sex) and some oral indices (teeth number, plaque index, gingival index, presence of gingivitis, lingual patina index, presence of oral infection, and oral hygiene) were significantly different among groups. Apart from sex, all these variables were significantly different between HC and patient groups, but they were not significantly different between ND and no-ND patients. All statistical results are shown in Table 2.

No significant difference was found in the presence of comorbidities (i.e., systemic inflammation, cholesterol level, triglycerides, hypertension, and diabetes) in the two patient groups. Table 3 shows the percentage of patients suffering from each comorbidity for the ND and no-ND groups with the statistical results in the comparison between the two groups of patients.

### 3.2. Pg Bacteria and Antibody Quantification

The Pg abundance in brushing sample was 8584 ±3139 (mean ± standard error) CFU/mL for the ND group, 1476 ± 731 CFU/mL for the no-ND group, and 1328 ± 708 CFU/mL for the HC. A significant difference was found among the groups in the abundance of Pg bacteria in brushing samples (F = 6.003, *p* = 0.004). Bonferroni’s post hoc test showed that Pg abundance was higher in ND patients than in no-ND patients (*p* = 0.010) and HC (*p* = 0.008). No significant difference was found between no-ND patients and HC (*p* = 1.00). All ND patients showed a detectable Pg quantity; 5 no-ND patients (18%) and 12 HC (41%) showed an absence of Pg in the brushing sample. The percentage of HC with a detectable Pg quantity in the oral cavity was higher than that of no-ND with a significant tendency (χ^2^ = 3.77, *p* = 0.052).

**Figure 1:**
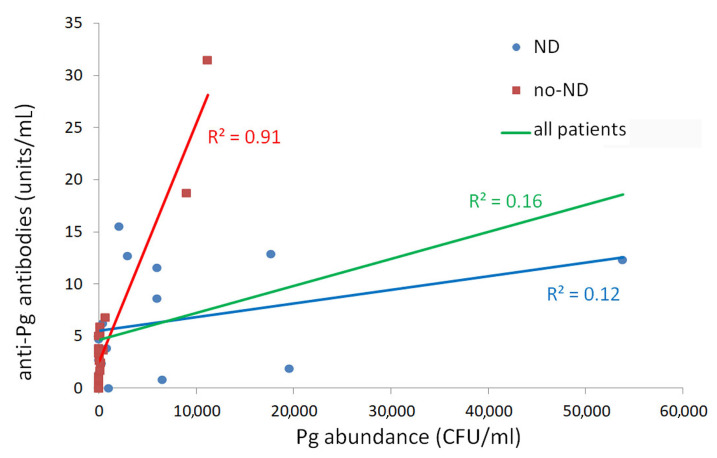
Relationship between anti-Pg antibodies and Pg abundance in all patients. Data were fitted with lines for ND, no-ND, and all patients.

No significant difference was found in anti-Pg antibodies abundance between ND and no-ND patients. One patient in the ND group and two patients in the no-ND group did not show detectable anti-Pg antibodies in the serum. Specifically, the anti-Pg antibodies quantity was higher in ND patients (6.52 ± 1.36 EU) than in no-ND patients (4.98 ± 1.58 EU), but this difference was not significant. A significant positive correlation was found between Pg bacteria and anti-Pg antibodies quantity for all patients (Spearman’s ρ = 0.54, *p* = 0.001). When the two patients’ groups were considered separately, the ratio between the anti-Pg antibodies quantity (in EU) and Pg abundance (in CFU/mL) was significantly higher in the no-ND than in the ND group (*p* = 0.037). This result is shown in Figure 1, where the slope of the straight line (indicating the ratio described above) is higher in the no-ND than in the ND group. In addition, no-ND data on Pg abundance in brushing and anti-Pg antibodies were fitted adequately by linear regression (R^2^ = 0.91); conversely, ND data were not fitted adequately by the linear regression (R^2^ = 0.12). These results evidenced that high levels of Pg in the oral cavity were associated with high serum IgG antibody levels against Pg in no-ND patients. Instead, in ND patients, a high Pg abundance in the oral cavity induced a low production of anti-Pg antibodies.

Because of the demyelination mechanism typical of MS pathology, which differentiates it from the other neurodegenerative diseases, a further subanalysis was performed excluding the two MS patients. The results of this analysis are shown in the Supplementary Materials.

No significant correlation was found between demographic and oral characteristics and Pg bacteria abundance in the oral cavity of all participants. No significant correlation was found between demographic and oral characteristics and anti-Pg antibodies quantity in the sera of the patients.

## 4. Discussion

Periodontal disease, or periodontitis, refers to the inflammatory processes that occur in the tissues surrounding the teeth in response to bacterial accumulations or dental plaque on the teeth. The bacterial accumulations cause a systemic inflammatory response. Although more than 700 bacterial species can colonize the oral cavity [47], only a handful of those are strongly implicated in periodontitis [48]. Pg is the species most strongly associated with the chronic form of periodontitis, and can be detected in up to 85% of the disease sites [49]. The influence of Pg on the onset of systemic inflammation has been confirmed by the finding that intensive periodontal therapy decreased levels of systemic inflammation [50,51]. Previous studies reported a relationship between Pg abundance and AD [52] or periodontal disease and AD [53,54], indicating a possible association between periodontal infection and AD [55]. Then, periodontitis was considered a risk factor for the worsening of neuroinflammation in AD progression [56,57], and it was recently associated with PD progression [58]. Thus, chronic inflammation seems to be an important factor in the process of neurodegeneration, causing dysregulation of circulating inflammatory molecules and the innate immune response [59].

We confirmed the relationship between Pg abundance in the oral cavity and neurodegenerative diseases. Patients suffering from a neurodegenerative disease showed a higher abundance of Pg in the oral cavity than patients affected by a neurological, non-degenerative disease and HC. Thus, our results extend our understanding of the association between oral bacteria and AD or PD, such as neurodegeneration, to other neurodegenerative diseases including parkinsonism, FTD, MS, and HD, indicating a new possible neuropathological pathway between periodontitis and neurodegenerative processes. The same results were also observed considering the ND group without MS patients, which are characterized by demyelination mechanisms.

Our results revealed a positive correlation between the abundance of Pg in the oral cavity and the quantity of anti-Pg antibodies in the serum of all patients, confirming the hypothesis that oral bacteria can cause a production of anti-Pg antibodies, as was reported previously [60]. In the present study, the anti-Pg antibodies production was not related to a clear systemic inflammatory process, as revealed by CRP, white WBC, NC, and lymphocytes count. The percentage of ND patients with systemic inflammatory process was not significantly different compared with no-ND patients (Table 3). When the analysis of anti-Pg antibodies was split for the ND and no-ND groups, the results for the no-ND patients showed a linear increase in anti-Pg antibodies quantity in serum versus Pg abundance in the oral cavity. This linear increase was not found in the ND group, where the greater abundance of Pg in the oral cavity was not linked to a higher anti-Pg antibodies quantity in serum. This result suggests that in patients affected by neurodegenerative disease, there is a low humoral immune response to periodontal pathogens, with the production of low antibodies quantities in serum. It is likely that the efficacy of the immune response affects the progression of the neurodegenerative disease, meaning that a lower or suppressed immune response, for example, due to immune kynurenines, could further progress the neurodegenerative disease.

Future studies on larger samples of ND and no-ND patients and on HC are required to confirm this hypothesis. At present, serum IgG antibodies against periodontal pathogens have been only associated with AD [30,31,32] and no quantitative association between Pg abundance and anti-Pg antibodies was previously reported.

The ND and no-ND patients had compromised oral health with low oral hygiene index, presence of oral infection, and reduced number of teeth. Tooth loss due to periodontal disease and irregular tooth brushing were associated with doubled AD risk [61] and higher dementia risk [62].

In addition to our cohort of patients and HC, the Pg abundance was not correlated with oral health indices, supporting previous results on Pg presence in periodontally healthy individuals [63,64]. This might be explained by the considerable strain diversity within the population structure of Pg. Moreover, key virulence factors of this pathogen (such as gingipains and lipid A phosphatases) are regulated by local environmental conditions, which may differ among individuals. In a related context, there might be individuals who can resist the conversion of a symbiotic microbiota into a dysbiotic one due to their intrinsic immune status; for example, they might have alterations in signaling pathways required for immune subversion by Pg or other keystone-like pathogens. The tongue surface is an important reservoir for periodontal pathogens such as Pg, which is associated with deep periodontal pockets and periodontal tissue inflammation [65]. PI values were significantly higher in ND and no-ND groups, both with high intraoral Pg, than in HC. Only ND patients, although showing similar PI values to no-ND patients, had significantly higher gingival inflammation and lingual patina than the HC, probably linked to their increased oral frailty [54,55,66,67]. The extraction of hopeless teeth, the treatment of oral infections, and wearing of dentures may have a beneficial effect on mastication and therefore on the reduction in dementia risk [55,56,68,69]. Pg has a number of methods to reach the brain from the infected periodontal pocket [70] and has been detected in the brain of animals and humans with AD [60,71]. Pg gingipains were implicated in the degradation of antibacterial peptides [72] and in the possible leakage of the bacterium [61,73]. Infection factors are among the ND-initiation factors, but they may also contribute to disease progression, according to the recently proposed cyclic potentiation between neurodegeneration and neuroinflammation [6].

The main limitations of the study are the small samples and the heterogeneity of the ND group. However, these preliminary results suggest that the same microbe (Pg) may be considered as the common neuropathological process among all different neurodegenerative diseases, such as in our sample. Despite each neurodegenerative disease being characterized by its own specific neuropathological process, neurodegenerative diseases share the activation of specific microglia and astrocytes-producing cytokines, chemokines, and other inflammatory molecules within the central nervous system. Activation of glial cells and astrocytes causes neuroinflammation, most probably the last common pathway leading to neurodegeneration [6]. Thus, we hypothesized that pro-inflammatory mediators may be also induced by Pg, promoting circulating inflammatory molecules.

However, further evaluations on a wider sample of ND patients are required to analyze possible differences among distinct neurodegenerative diseases in the systemic responses to oral pathogens. Furthermore, future studies on OMVs will help to clarify the possible passage of Pg through the blood–brain barrier.

## 5. Conclusions

This study’s findings suggest a possible bidirectional oral–brain axis in which oral pathogens can induce a system response and neurodegenerative processes can reduce the production of anti-oral pathogen antibodies, with a possible negative impact on disease progression. Even if the risk of periodontal disease for the progression of neurodegenerative diseases needs to be further substantiated, it is advisable to implement prevention through periodontal prophylaxis, intensive periodontal therapy, and therapeutic interventions.

## Figures and Tables

**Table 1:** Demographic characteristics and oral indices of all participants of the groups included in the study. Otherwise, the number of patients included in the calculation is shown in parentheses.

Variables	ND Patients (*n* = 21)	No-ND Patients (*n* = 28)	HC (*n* = 29)
Age, years (mean ± SD)	70.6 ± 13.4	67.6 ± 19.8	56.1 ± 12.8
Sex (% male)	66.7	28.6	44.8
Teeth number (mean ± SD)	15.8 ± 8.8	14.6 ± 10.2	22.5 ± 6.3
Plaque index (mean ± SD)	2.1 ± 0.9 (*n* = 8)	2.0 ± 0.3 (*n* = 8)	1.2 ± 0.6
Gingival index (mean ± SD)	1.5 ± 0.8 (*n* = 8)	1.1 ± 0.5 (*n* = 8)	0.6 ± 0.5
Presence of gingivitis (% of pts)	88	69	52
Lingual patina index (mean ± SD)	1.6 ± 1.3 (*n* = 16)	0.8 ± 0.9 (*n* = 25)	0.4 ± 0.5 (*n* = 14)
Presence of oral infection (% of pts)	82.4 (*n* = 17)	64 (*n* = 25)	41.4
Oral hygiene index (mean ± SD)	0.2 ± 0.4 (*n* = 17)	0.4 ± 0.6 (*n* = 25)	1.2 ± 0.6
Presence of fissured tongue (% of pts)	5.9 (*n* = 17)	7.7 (*n* = 26)	1
Number of fixed prosthesis (mean ± SD)	0.3 ± 0.7	0.4 ± 0.7	0.4 ± 0.6
Number of removable denture (mean ± SD)	0.5 ± 0.7	0.5 ± 0.8	0.2 ±0.5
Smoker (% of pts)	0	11.1 (*n* = 27)	20.7
Former smoker (% of pts)	28.6	11.1 (*n* = 27)	20.7

**Table 2:** Statistical results of the comparison of demographic characteristics and oral indices among groups included in the study. Significant results are highlighted in bold.

	Main Effect	Post-Hoc ND vs. no-ND	Post-Hoc ND vs. HC	Post-Hoc No-ND vs. HC
Age (mean ± st.dev.)	F = 6.200, ***p* = 0.003**	*p* = 1	***p* = 0.006**	***p* = 0.022**
Sex	***p* = 0.031**	***p* = 0.025**	*p* = 0.384	*p* = 0.661
Teeth number	F = 6.866, ***p* = 0.002**	*p* = 1	***p* = 0.022**	***p* = 0.003**
Plaque index	***p* = 0.001**	*p* = 1	***p* = 0.013**	***p* = 0.004**
Gingival index	***p* = 0.005**	*p* = 1	***p* = 0.008**	*p* = 0.155
Presence of gingivitis	***p* = 0.040**	*p* = 0.598	***p* = 0.035**	*p* = 0.516
Lingual patina index	***p* = 0.015**	*p* = 0.131	***p* = 0.016**	*p* = 0.607
Presence of oral infection	***p* = 0.006**	*p* = 0.704	***p* = 0.004**	***p* = 0.050**
Oral hygiene index	***p* = 0.001**	*p* = 0.967	***p* = 0.001**	***p* = 0.001**
Presence of fissured tongue	*p* = 0.675			
Number of fixed prosthesis	*p* = 0.590			
Number of removable denture	*p* = 0.132			
Smoker	*p* = 0.082			
Former smoker	*p* = 0.315			

**Table 3:** Presence of comorbidities and statistical results in the comparison between the two groups of patients.

Patients (%)	ND (*n* = 21)	No-ND (*n* = 28)	Statistical Results
Systemic Inflammation	56	52	Χ^2 =^ 0.06 *p* = 0.80
Cholesterol ratio > cut-off	20	44	Χ^2 =^ 1.53 *p* = 0.22
High triglycerides	20	38	Χ^2 =^ 0.89 *p* = 0.35
Hypertension	39	56	Χ^2 =^ 1.20 *p* = 0.27
Diabetes	22	26	χ^2 =^ 0.08 *p* = 0.78

## Supplementary Materials

The following are available online at https://www.mdpi.com/article/10.3390/biom11060845/s1.
