# Supplementary material for: Exploring the Connection between Porphyromonas gingivalis and Neurodegenerative Diseases: A Pilot Quantitative Study on the Bacterium Abundance in Oral Cavity and the Amount of Antibodies in Serum
Source: Biomolecules. 2021 Jun 6;11(6):845. doi: 10.3390/biom11060845 (PMC8229521; doi:10.3390/biom11060845)
Supplement: Supplementary file 1 [file biomolecules-11-00845-s001.zip › biomolecules-1229006-supplementary.pdf]

Supplementary Material

As a second analysis we excluded from the ND group the two patients affected by MS and we performed the statistical comparisons among the three groups as previously done. The statistical results are similar to the ones which were described in the main text. Specifically, the Pg abundance in brushing sample was 8146±3423 (mean±standard error) CFU/ml for ND group, 1476±731 CFU/ml for no-ND group and 1328±708 CFU/ml for HC. Significant difference was found among the groups in the abundance of Pg bacteria in brushing samples (F=4.914, p=0.010). Bonferroni post-hoc showed that Pg abundance was higher in ND patients than no-ND patients (p=0.022) and HC (p=0.017). No significant difference was found between no-ND patients and HC (p=1.00).

No significant difference was found on anti-Pg antibody abundance between ND patients and no-ND patients. Specifically, the anti-Pg antibodies quantity was higher in ND patients (6.71±1.52 EU) than no-ND patients (4.98±1.58 EU), but this different was not significant (p=0.47). A significant positive correlation was found between Pg bacteria and anti-Pg antibodies quantity on all patients (Spearman ρ=0.58, p=0.0003). When the two patients' groups were considered separately, the ratio between the anti-Pg antibody quantity (in EU) and Pg abundance (in CFU/ml) was higher significantly in no-ND than ND group (p=0.040).

Also with the exclusion of the two patients affected by MS, ND data were not fitted adequately by the linear regression, showing R^2= 0.19 and confirming that a great Pg abundance in the oral cavity induces a low production of anti-Pg antibodies in ND patients.
